# Characterizing Visual Field Defects with Tangent Screen Perimetry in Organic Versus Non-Organic Pathologies

**DOI:** 10.3390/diagnostics16060842

**Published:** 2026-03-12

**Authors:** Hyunmin Na, Jeong-Min Hwang, Hee Kyung Yang, Sang Beom Han

**Affiliations:** 1 Department of Ophthalmology, Seoul National University College of Medicine, Seoul National University Hospital, Seoul 03080, Republic of Korea; nhmiswow@naver.com; 2 Department of Ophthalmology, Kim’s Eye Hospital, Seoul 07301, Republic of Korea; 3 Department of Ophthalmology, Seoul National University College of Medicine, Seoul National University Bundang Hospital, Seongnam 13620, Republic of Korea; 4 Saevit Eye Hospital, Goyang 10447, Republic of Korea

**Keywords:** tangent screen perimetry, organic visual loss, functional visual loss

## Abstract

**Background/Objectives:** Tangent screen perimetry is a valuable tool for detecting functional visual loss (FVL), which is suspected when the visual field fails to expand as expected with distance. However, there is currently a lack of research documenting the specific tangent screen patterns produced by patients with organic visual loss (OVL), defined as visual field loss caused by identifiable structural or neurologic pathology. This study aims to characterize the visual field patterns observed in patients with organic and functional pathologies during tangent screen perimetry and evaluate its diagnostic efficacy in confirming FVL. **Methods:** Medical records of patients from Seoul National University Bundang Hospital between August 2009 and August 2019 were reviewed. All subjects underwent a comprehensive neuro-ophthalmologic examination with additional testing to confirm the diagnosis of OVL or FVL. A total of 126 eyes from 76 patients exhibiting visual field constriction within 30 degrees were included. The tangent ratio (TR) was defined as the average visual field (in radians) at a far distance (e.g., 2 m) divided by the average visual field at a near distance (e.g., 1 m). The visual field patterns and TR were analyzed, and the diagnostic value of TR in detecting FVL was determined. **Results**: The clover leaf pattern and reversal pattern were observed in 8.8% and 12.7% of FVL cases, respectively, whereas no such patterns were found in OVL cases (*p* = 0.002, *p* < 0.001). The TR varied from 0.50 to 1.06 (mean 0.77 ± 0.16) in OVL and from 0.33 to 1.03 (mean 0.65 ± 0.15) in FVL (*p* < 0.001). Younger age, a clover leaf pattern or reversal pattern on tangent screen perimetry, and a lower TR were significantly associated with FVL. **Conclusions**: Tangent screen perimetry is an effective adjunct for differentiating functional from organic visual field loss, particularly in cases of visual field constriction.

## 1. Introduction

Patients may present with visual complaints that cannot be fully explained by identifiable structural abnormalities of the eye or visual pathways. This clinical entity, broadly referred to as functional visual loss (FVL), encompasses a heterogeneous spectrum of psychogenic, attentional, and neurocognitive mechanisms rather than a single diagnostic category or deliberate exaggeration alone [1,2,3,4]. Contemporary studies emphasize that FVL frequently coexists with organic visual pathology, further complicating diagnostic interpretation and increasing the risk of misclassification, particularly in medico-legal, occupational, or disability-related contexts [5,6,7,8]. Accordingly, functional and organic visual field constriction should be viewed as overlapping, rather than mutually exclusive, diagnostic categories [9]. Notably, current clinical understanding clearly distinguishes functional visual loss from malingering, recognizing that intentional symptom exaggeration represents only a minority of cases, while most patients exhibit involuntary or unconscious visual dysfunction [3,4]. Longitudinal studies have reported that approximately 25% to 78% of patients with functional visual loss show partial or complete symptom improvement during follow-up [10,11].

Misdiagnosis may lead either to unnecessary investigations and interventions or, conversely, to delayed identification of underlying neurologic disease, underscoring the importance of reliable diagnostic adjuncts [12,13,14]. Accurate diagnosis and clear communication at the initial encounter may play a central role in determining clinical outcomes in patients with functional neurological symptoms [15].

Visual field testing remains one of the most widely used objective tools in the evaluation of suspected FVL, as well as organic visual loss (OVL), which is defined as visual impairment attributable to identifiable structural or neurologic pathology affecting the visual system. In both entities, perimetric assessment is routinely employed to characterize the extent and pattern of visual field deficits, despite fundamental differences in their underlying mechanisms. However, test reliability is highly variable, and nonphysiologic response patterns have been reported not only in patients with FVL but also in those with confirmed organic disease or cognitive impairment [16,17,18].

In some cases, the visual field test may show inconsistencies, such as large variations in the area of the visual field [19,20,21]. Specific signs, such as a spiral defect or crossing visual field in the Goldmann visual field test, suggest FVL [18,22,23,24]. Such configurations have traditionally been regarded as nonphysiologic because they violate the geometric behavior expected of true retinal or post-chiasmal defects. The most common type of visual field defect in FVL is concentric peripheral loss, also known as tunnel vision. This pattern is particularly problematic because it may also be encountered in certain neurologic or retinal disorders, thereby representing a major source of diagnostic ambiguity. In these cases, the tangent screen is used to determine if the patient’s visual field expands proportionally as the distance from the target is increased. The test is based on the principle that the angular extent of the visual field should remain constant when projected. This physiologic expansion is well-documented in healthy adults, as demonstrated in our previous prospective experimental study [25]. In visually intact subjects, this distance-dependent scaling follows a near-linear relationship under controlled conditions. FVL is suspected when the visual field fails to expand as expected with distance, does not expand at all, or even appears to shrink [5,22,26,27,28,29]. Such paradoxical behavior is considered incompatible with known optical or neuroanatomical impairments. In contrast, organic visual field defects should follow the laws of optics, expanding linearly as the distance from the tangent screen increases. This assumption has historically formed the conceptual basis for using tangent screen perimetry as a confirmatory adjunct in suspected functional visual loss. Nevertheless, tangent screen findings should not be interpreted as a standalone or binary discriminator between functional and organic visual loss; rather, they should be considered within the broader clinical, neurologic, and imaging context as one component of a multimodal diagnostic assessment.

However, there is currently a lack of research documenting the specific tangent screen patterns produced by patients with definitive organic visual field defects. Accordingly, the present study was designed to address these gaps by systematically characterizing visual field patterns observed during tangent screen perimetry in patients with independently confirmed organic and functional visual loss. In addition, we evaluated the diagnostic performance of a quantitative tangent ratio (TR) and to evaluate its diagnostic efficacy in confirming functional visual loss.

## 2. Materials and Methods

### 2.1. Subjects

The medical records of subjects between August 2009 and August 2019 were retrospectively reviewed from Seoul National University Bundang Hospital. The medical history of all subjects was taken, and they all underwent complete ophthalmic examination, including visual acuity assessment, refractive error assessment, slit-lamp biomicroscopy, presence of strabismus assessment, intraocular pressure assessment, and fundoscopy. Subjects underwent additional exams, if needed, such as electroretinogram (ERG) (VERIS II; ElectroDiagnostic Imaging 45 Inc., San Francisco, CA, USA), fluorescein angiography (FAG) (VX-10; Kowa OptiMed, Tokyo, Japan), indocyanine green angiography (ICGA) (Heidelberg Retina Angiography; Heidelberg Engineering, Heidelberg, Germany), optical coherence tomography (OCT) (SD-OCT; Spectralis OCT, Heidelberg, Engineering, Heidelberg, Germany), OCT angiography (OCTA) (DRI-OCT Triton, Topcon, Tokyo, Japan), red-free fundus imaging (EOS D60 digital camera; Canon, Utsunomiyashi, Tochigiken, Japan), and magnetic resonance imaging/magnetic resonance angiography (MRI/MRA) under a physician’s discretion. These additional examinations were selectively performed only when clinically indicated to exclude or confirm an underlying organic cause of visual field constriction. All subjects underwent Goldmann perimetry (Haag-Streit, Bern, Switzerland) or standard automated perimetry (24-2 Swedish interactive threshold algorithm and Humphrey Field Analyzer II 750, Carl Zeiss Meditec, Jena, Germany) and a tangent screen test. The diagnosis of organic disease was retrospectively confirmed by three of the authors independently, where two of the authors are neuro-ophthalmology specialists, and corroborating evidence was provided through ancillary testing, including the above-mentioned ophthalmic tests or imaging studies, which was read by a neuroradiologist. Note that the diagnosis was made without tangent screen testing. This study was approved by the Institutional Review Board of Seoul National University Bundang Hospital, and it conformed to the tenets of the Declaration of Helsinki.

### 2.2. Tangent Screen Perimetry

A black felt screen was placed with a pattern of circles and meridians sewn in black. A white button was at the center. A subject was seated one meter from the screen, and their eye level and center of the screen were set at the same height. The lights were dimmed with one eye occluded. The subject was indicated not to rotate or move their neck or move their eyes. Then, the subject was instructed to fixate on the central white button. Starting with the top center radian, the examiner moved the object slowly towards the center. At the specific point where the object was recognized by the subject, a black pin was inserted into the screen to mark the maximal visual field on that meridian. This process was repeated for each meridian. When the visual field of the subject covered less than half of the largest circles, the subject was seated two meters away from the screen and the process was repeated. If the visual field at two meters exceeded the limit of the test, the patient was seated 50 cm away from the screen and the process was repeated. When the visual field of the subject at one meter covered more than half of the screen, the patient was seated 50 cm away from the screen and the process was repeated. In a similar manner, the process was completed when the constricted visual fields of two consecutive distances were obtained (i.e., 25 cm–50 cm, 50 cm–1 m, or 1 m–2 m).

### 2.3. Eligibility Criteria

Study participants included individuals aged 6 to 85 years with central 30-degree field defects identified via automated or manual perimetry, all of whom underwent supplementary tangent screen analysis. Exclusion criteria included patients showing no field constriction on tangent screen perimetry despite the presence of visual field constriction on Goldmann field perimetry or Humphrey field perimetry, poor visual acuity, poor cooperation in tangent screen perimetry, and unclear diagnosis even after thorough examinations. All included patients underwent comprehensive neuro-ophthalmologic evaluation, including best-corrected visual acuity assessment, intraocular pressure measurement, ocular alignment assessment, fundus examination, optical coherence tomography, and additional testing as needed to establish a definitive diagnosis of organic or functional visual loss.

### 2.4. Outcome Measures

**Figure 1:**
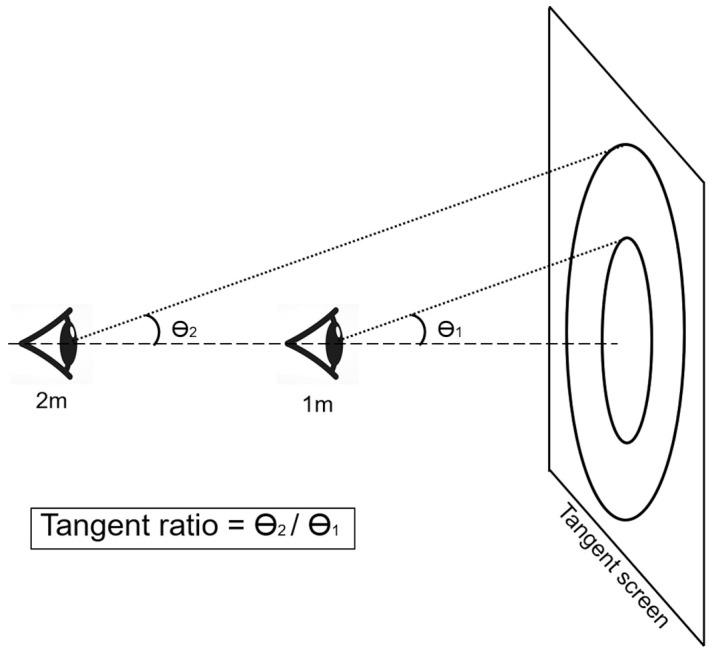
Definition of tangent ratio.

The primary outcome measure in this study was TR (Figure 1), defined as the averaged visual field (radian) at a far distance (e.g., 2 m) over the averaged visual field (radian) at a near distance (e.g., 1 m). The averaged visual field was calculated by taking the average of the maximal radians as the subject recognized on each meridian. For example, if one showed temporal hemianopsia, the mean value was calculated by averaging the radians of the superior, superonasal, nasal, inferonasal, and inferior meridians. To convert linear distances on the tangent screen to angular visual field values, the TR was calculated as follows:

Tangent ratio (TR) = arctan(d_far/D_far) ÷ arctan(d_near/D_near), where d_far and d_near represent the linear distances from the fixation center to the recognized target at the far and near distances, respectively, and D_far and D_near denote the corresponding screen distances (e.g., 2 m and 1 m).

**Figure 2:**
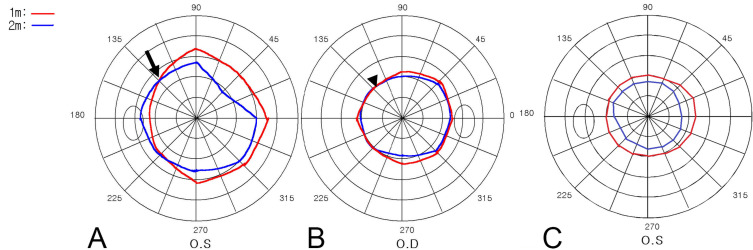
Patterns of visual field testing on tangent screen perimetry in patients with functional visual loss. Red lines indicate the visual field measured at 1 m, and the blue lines indicate the visual field measured at 2 m. (**A**) The black arrow indicates the intersection of the visual field. (**B**) The black arrowhead indicates overlapping in the visual field. (**C**) Shrinkage of the visual field. Note that the visual field at 1 m is larger than at 2 m.

Secondary outcome measures included whether a subject showed a clover leaf pattern or intersecting (Figure 2A) or overlapping (Figure 2B) visual fields at two distances, or visual field shrinkage (Figure 2C).

Abnormal tangent screen patterns are defined as follows:

An overlapping pattern was defined as a qualitative pattern in which the visual field areas obtained at near (1 m) and far (2 m) distances appeared similar in size and largely overlapped, showing little or no expected expansion with an increasing test distance. An intersecting pattern was defined when the visual field contours at two distances crossed each other on at least one meridian. A reversal pattern (shrinkage pattern) was defined when the visual field measured at the near distance was consistently smaller than that measured at the far distance, indicating paradoxical shrinkage with proximity.

We also investigated the diagnostic value of tangent screen perimetry in patients with visual field loss.

### 2.5. Statistical Analysis

Statistical analyses were performed using the Statistical Package for the Social Sciences (version 22.0, SPSS, Chicago, IL, USA). Except where stated otherwise, the data are presented as mean ± SD values, and a *p* value less than 0.05 was accepted as significant. Significant differences between OVL and FVL were evaluated using Levene’s homogeneity of variance test and an independent *t*-test. To address the potential non-independence arising from the inclusion of both eyes from the same participant, we performed an additional mixed-effects analysis for the TR, which was the primary quantitative outcome. Specifically, a linear mixed-effects model was fitted with the TR as the dependent variable, group (FVL vs. OVL) as a fixed effect, and subject (patient identification number) as a random intercept, thereby accounting for within-subject correlation regarding subjects’ eyes. Parameters were estimated using restricted maximum likelihood, and the results are reported as regression coefficients (β) with standard errors and 95% confidence intervals (two-sided tests; *p* < 0.05).

## 3. Results

**Figure 3:**
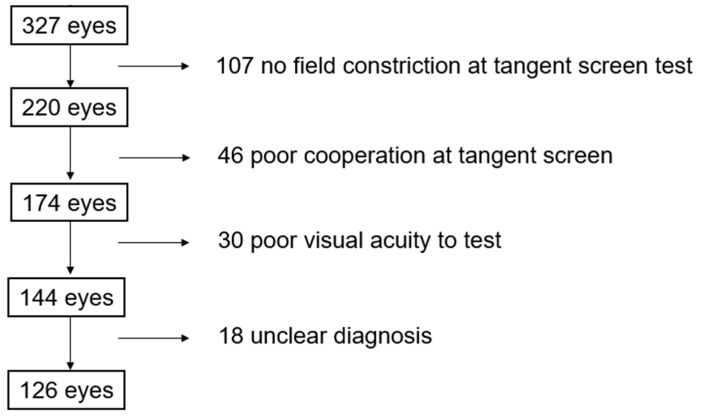
Inclusion criteria of the study cohort.

A retrospective review was performed on 327 eyes of 165 patients, which showed visual field constriction within 30 degrees on Goldmann perimetry or with the Humphrey visual field analyzer between 1 August 2009 and 1 August 2019. After excluding patients who failed to meet the eligibility requirements, 126 eyes of 76 patients (41 males) were analyzed (Figure 3). This stepwise selection process ensured that only patients with consistent evidence of visual field constriction and reliable tangent screen testing were included in the final analysis.

The age of patients varied from 6 to 85 (28.48 ± 19.00). Visual acuities of patients showed a wide range from −0.301 to 1.854 logMAR (0.631 ± 0.569). These wide age and visual acuity distributions reflect the heterogeneous clinical settings in which visual field constriction may be encountered and underscores the need for a simple screening tool applicable across a diverse patient population. Among the 76 patients, 26 (34.2%) were pediatric (<18 years), contributing 47 of 126 eyes (37.3%). The majority of pediatric patients contributed both eyes (21/26, 80.8%). Within this subgroup, 16 patients were aged 6–11 years, while 10 patients were aged 12–17 years. Among 126 eyes, 24 eyes (19%) were diagnosed with OVL, and 102 eyes (81%) were diagnosed with FVL. Causes of visual loss in the OVL group were stroke, retinitis pigmentosa, brain tissue loss, brain tumor, glaucoma, and optic neuropathies. (Table 1) This etiologic diversity indicates that the organic group represented a broad spectrum of retinal and neurologic disorders.

None (0%) of the patients with OVL and 9 eyes (8.8%) of patients with FVL showed a clover leaf pattern (*p* = 0.002). Two eyes (8.3%) of patients with OVL and seventeen eyes (16.7%) of patients with FVL showed either overlapping or intersecting visual fields. A reversal pattern was found in 13 eyes (12.7%) of patients with FVL, while there was no such case in patients with OVL (*p* < 0.001). These results indicate that paradoxical or non-physiologic tangent screen patterns are strongly suggestive of functional pathology and are rarely observed in organic disease.

Significant factors predicting FVL included younger age, the presence of a clover leaf or reversal pattern, and a lower TR. The reversal pattern in tangent screen perimetry was the most powerful predictor of FVL. None of the OVL patients exhibited a reversal pattern, while 12.7% of FVL patients did.

**Figure 4:**
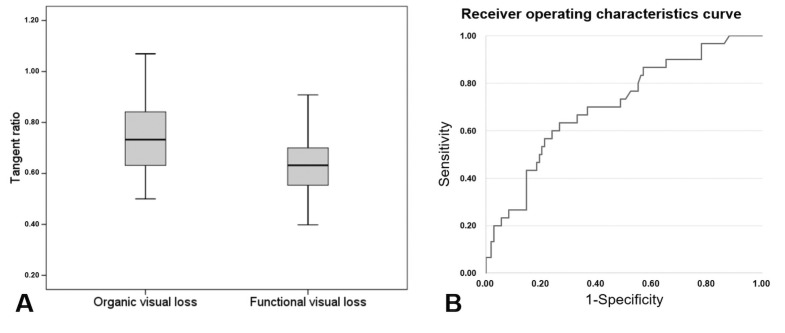
Tangent ratio in organic and functional visual loss. (**A**) Box-plot of tangent ratio. (**B**) Receiver operating characteristics curve with respect to tangent ratio.

The TR varied from 0.50 to 1.06 (0.77 ± 0.16) in OVL patients and 0.33 to 1.03 (0.65 ± 0.15) in FVL patients, as shown in Figure 4A. The lower TR values observed in FVL patients reflect a failure of the visual field to expand proportionally with increasing distance, which is a classic feature of functional visual loss. Figure 4B shows the receiver operating characteristics curve of FVL patients with respect to the TR. The area under curve was 0.725, the optimal cutoff point of the TR was 0.64, and the sensitivity and specificity were 87.5% and 50.0%, respectively. Table 2 shows the diagnostic accuracy of tangent screen perimetry with respect to various cutoff points of the TR. With a cutoff value of TR < 0.7, the sensitivity of tangent screen perimetry to detect FVL is 62.5% with a specificity rate of 67.6%.

Given that most participants contributed both eyes, we performed an additional linear mixed-effects model to account for within-subject correlation. This approach allowed us to appropriately model the non-independence of paired eyes while preserving the full dataset. In this model, the TR was the dependent variable, the group (FVL vs. OVL) was included as a fixed effect, and the subject (patient identification number) was included as a random intercept. The mixed-effects model confirmed that the TR remained significantly lower in FVL patients than in those with OVL (β = −0.107, SE = 0.038, *p* = 0.005), corresponding to an estimated mean difference of approximately 0.11 after accounting for paired-eye clustering (95% CI, −0.182 to −0.032). Notably, the intraclass correlation coefficient was high (ICC = 0.67), indicating substantial within-subject correlation between fellow eyes and supporting the appropriateness of the mixed-effects approach.

**Figure 5:**
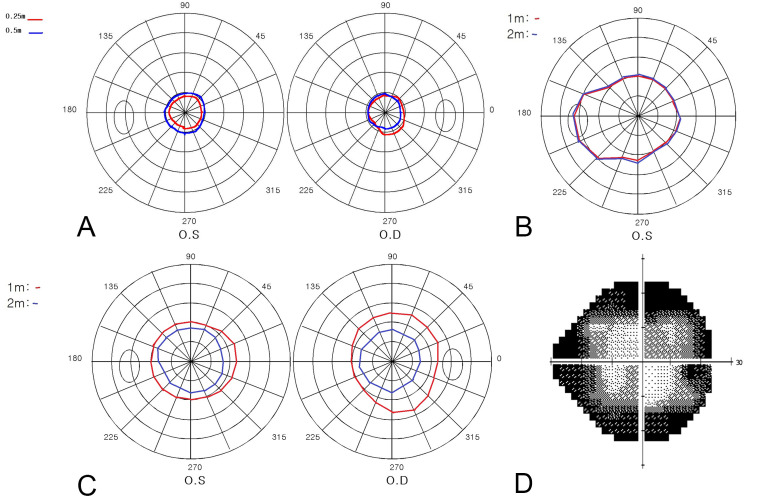
Specific patterns of visual field testing in functional visual loss. (**A**) Intersecting, (**B**) overlapping, (**C**) reversal, and (**D**) clover leaf patterns.

Specific patterns of visual field were more frequently found in FVL patients. Figure 5 shows examples of visual field types in FVL patients. These representative cases visually demonstrate the paradoxical behavior of functional visual fields across different clinical contexts. Figure 5A shows the intersecting pattern of a malingering patient. Figure 5B shows the case of a patient following blunt head trauma after a traffic accident, showing an overlapping pattern with no definite organic disease after thorough ophthalmic and neurologic examinations. Figure 5C shows a reversal pattern of a patient with anxiety disorder. Figure 5D shows the clover leaf pattern observed in a patient with non-organic visual field constriction.

## 4. Discussion

Tangent screen perimetry is a valuable tool in detecting functional visual loss. Previous studies have suggested that the visual field should remain consistent with the test distance. In cases where the visual field either does not expand or even shrinks with distance, FVL is suspected [30,31]. In our earlier study [25], we found that the visual field in normal adults did not change with the test distance with a normal TR of 0.96 ± 0.1. No cases showed a TR less than 0.50, indicating that there were no instances of visual field shrinking or intersection in normal adults.

In this study, we examined the TR of patients with OVL. The TR in the OVL group (0.77 ± 0.16) was larger than in the FVL group, but lower than the health controls in our previous study (0.96 ± 0.1.). Predictors of FVL were a clover leaf pattern or reversal pattern on tangent screen perimetry, a lower TR, and younger age.

There was no correlation between visual acuity and TR in OVL patients (*p* = 0.880), while better visual acuity was associated with a larger TR in FVL patients (*p* = 0.014). Interestingly, the TR in the OVL group (0.77 ± 0.16) was lower than expected, though it was still significantly different from that in the FVL group (0.65 ± 0.15, *p* < 0.001). One possible explanation for this discrepancy is the large selection bias in the OVL group. Since the patients in this study were selected based on the suspicion of FVL, the OVL group may have included more cases with exaggerated or unusual visual field loss, which could account for the lower-than-expected TR. This finding further supports the notion that functional and organic visual field constriction represent overlapping clinical spectra rather than mutually exclusive categories. This concept is in line with the contemporary framework of functional neurological disorders, in which symptoms are understood as genuine perceptual experiences occurring despite preserved structural integrity of the visual pathways [32]. Furthermore, despite definitive diagnoses, the potential for malingering or secondary gain cannot be entirely excluded in certain cases. However, functional visual loss should not be equated with malingering, as non-organic visual field constriction may also arise from involuntary psychogenic, attentional, or neurocognitive mechanisms.

Additionally, we found that abnormal visual patterns, such as the clover leaf, intersecting, overlapping, and reversal patterns, were significant indicators of FVL. While none of the OVL patients showed the clover leaf pattern, 8.8% of FVL patients did (*p* = 0.002). Furthermore, intersecting or overlapping visual patterns were observed in 16.7% of FVL cases, compared to 8.3% in OVL cases, though the difference was not statistically significant (*p* = 0.230). Reversal patterns were found exclusively in FVL cases, with 12.7% of FVL patients showing this pattern (*p* < 0.001). This suggests that the presence of a reversal pattern is a strong indicator of FVL. On the contrary, given that only 38% of patients with functional visual loss exhibited abnormal patterns, the absence of such findings is insufficient to confirm organic visual loss. Consequently, the tangent screen should be viewed as a tool for confirmation rather than exclusion.

Age was also an important factor. Younger patients (under 45 years) were more likely to have FVL, while older patients were more likely to have OVL. Specifically, 91% of patients in the younger group (<45 years) had FVL, while only 48% in the older group (≥45 years) had FVL. It is therefore more appropriate to interpret these age-related differences as reflecting varying underlying mechanisms, with younger patients more likely to exhibit functional visual field constriction and older patients more likely to harbor organic pathology.

Some limitations should be acknowledged when interpreting the findings of this study. First, the retrospective design inherently introduces selection bias. All patients were referred for tangent screen perimetry based on a clinical suspicion of functional visual loss, which may have enriched the cohort with atypical or exaggerated visual field patterns. Consequently, the distributions of TR and visual field configurations observed in both the OVL and FVL groups may not fully represent unselected populations presenting with visual field constriction. Second, although Goldmann perimetry and automated visual field testing were used to define visual field constriction, the classification of FVL and OVL ultimately relied on clinical judgment and available medical records. Subclinical or early organic pathology may not have been completely excluded in all cases. Conversely, some patients categorized as OVL may have had a functional overlay, reflecting the complex and often mixed nature of visual field loss in real-world clinical settings. Third, tangent screen perimetry is inherently examiner-dependent and subject to variability related to patient understanding, cooperation, fatigue, and attentional factors. The manual recording of responses and visual field boundaries may introduce measurement error, particularly in pediatric patients or in those with cognitive or psychiatric comorbidities. Although all examinations were performed by experienced clinicians, inter- and intra-observer variability could not be formally assessed in this study. Fourth, this study was cross-sectional and did not evaluate longitudinal changes in the TR or visual field patterns.

Despite these limitations, the present study provides clinically meaningful evidence that tangent screen perimetry, particularly the TR and reversal pattern, can serve as a practical adjunct in differentiating functional from organic visual field constriction. Prospective, longitudinal studies with standardized diagnostic criteria and quantitative perimetric protocols are warranted to further validate and refine these findings.

## 5. Conclusions

While our previous research established that normal adults exhibit physiologic expansion of the visual field relative to distance, this study characterizes the performance of patients with organic visual loss (OVL) compared to those with functional visual loss (FVL). Although a statistically significant difference in TR was observed between the two groups, the TR in the organic group was lower than theoretically expected, resulting in considerable overlap. Tangent screen perimetry remains a beneficial diagnostic adjunct when differentiating organic from functional pathology, particularly through the analysis of TR values and specific patterns, such as the ‘cloverleaf’ or ‘reversal’ patterns. However, we emphasize that tangent screen perimetry should be utilized as a supplementary tool rather than a definitive diagnostic test for differential diagnosis.

## Figures and Tables

**Table 1:** Demographics and characteristics of patients with visual field constriction due to organic versus functional visual loss.

	Organic Visual Loss (24 Eyes)	Functional Visual Loss (102 Eyes)	*p* Value
Age	45.9 ± 19.6	24.6 ± 16.7	<0.001
Sex (male)	15 (63%)	53 (52%)	0.355
Laterality (right)	13 (54%)	50 (49%)	0.653
BCVA (logMAR)	0.79 ± 0.65	0.59 ± 0.53	0.184
Clover leaf	0 (0%)	9 (8.8%)	0.002
Intersecting or overlapping	2 (8.3%)	17 (16.7%)	0.230
Reversal	0 (0%)	13 (12.7%)	<0.001
Tangent ratio	0.77 ± 0.16	0.65 ± 0.15	<0.001
Cause of disease *	Retinitis pigmentosa (4) Stroke (7) Brain tissue loss (4) Brain tumor (2) Glaucoma (1) Optic neuritis (1) Optic atrophy (5)	Not applicable	

BCVA = best-corrected visual acuity; logMAR = Logarithm of the Minimum Angle of Resolution. * Number of eyes.

**Table 2:** Diagnostic accuracy of the tangent ratio in diagnosing functional visual loss.

Cutoff Point	Sensitivity	Specificity	PPP	NPP
0.5	1.000	0.167	0.220	1
0.6	0.875	0.382	0.250	0.929
0.7	0.625	0.676	0.313	0.885
0.8	0.375	0.824	0.333	0.848
0.9	0.292	0.922	0.467	0.847
1	0.083	0.990	0.667	0.821

PPP = positive predictive power, and NPP = negative predictive power.

## Data Availability

The data supporting the findings of this study are not publicly available due to privacy and ethical restrictions, as they contain information that could compromise the privacy of research participants. Data are available from the corresponding authors upon reasonable request and with permission from the institutional review board.
